# Housing quality and its impact on Acute Respiratory Infection (ARI) symptoms among children in Punjab, Pakistan

**DOI:** 10.1371/journal.pgph.0000949

**Published:** 2022-09-21

**Authors:** Ammar Aftab, Amamah Noor, Memuna Aslam

**Affiliations:** School of Humanities and Social Sciences, Information Technology University, Lahore, Punjab, Pakistan; PLOS: Public Library of Science, UNITED STATES

## Abstract

Exposure to poor housing quality and household air pollution (HAP) are significant factors for morbidity and mortality in Pakistan. Children under five are particularly at risk of acute respiratory infections (ARIs). Globally, it has been estimated that 6.6 million children less than five years of age die every year from this disease. Little is known regarding the effect of HAP and poor housing quality on children’s respiratory symptoms in Pakistan. Statistics concerning Pakistan’s largest province, Punjab, are also not promising. Hence, an association between housing quality and respiratory symptoms among children under the age of five in Punjab has been studied in this paper. Using data from MICS 2017–18, logistic regression models were applied to the sample of 35000 children under the age of five living in poor housing quality. We estimated that acute respiratory infection (ARI) symptoms are higher among children when the floor of a house is made of natural material. However, the lower ARI symptoms were found among children living in a house with a wall made of natural material. On the other hand, we found that children residing in the western region of Punjab are at a higher risk of ARI symptoms. The findings remain consistent with the previous researchers. In addition to promoting increased access to quality housing material during construction activities, we suggest that critical community-based interventions are required to combat local issues and problems at the micro-level.

## Introduction

Housing quality is considered one of the most critical components of sustainable development in many countries. It shapes the physical accommodation and the health of a community, the social well-being of the individual and family, and the mental health of the occupants [1]. By definition, poor housing quality (durable structures) refers to dwellings that have dirt (earth), sand, mud or dung floors [2]. Rapid urbanisation can lead to many slum dwellers, inadequate infrastructure and services, improper water, and sanitation systems, worsening air pollution and unplanned urbanisation sprawl [2]. It is estimated that 3 billion people will require adequate housing by 2030 [3].

Many past researchers have used the risk factor as indoor air pollution from extensive usage of solid fuels, but now the studies use the term “household air pollution” that describes both the health-damaging exposures from indoor and outdoor cooking and the leakage of cooking activities into the neighbouring areas of the home [4]. According to the US’s Environment Protection Agency (EPA), an essential factor in determining indoor air pollutant concentrations in the air exchange rate with the outdoors. The exchange rate of air gets affected by the construction, design, and operating parameters of buildings. The air exchange rate is eventually a direct function of infiltration (air flowing into structures through cracks, openings, and joints in floors, walls, ceilings, doors, and windows), natural ventilation, and mechanical ventilation.

It is also essential to highlight the increasing housing and environmental-related health problems in developing countries as they are experiencing a rapid rate of urbanisation [5]. Pakistan is a developing country with one of the highest urbanisation rates in South Asia [6]. However, the government has not provided adequate and safe housing for low-income citizens. In Pakistan, 68% of the urban population from low-income households lives on 1% of the city’s land; in comparison, 23% of the people from middle-income families live on 43% of the land, whereas 12% of the upper-income household population lives on 56% of the land, and 47% of urban Pakistani lives in poor quality of houses whereas this results in increasing inequality in a country [7]. In terms of housing materials, the most common ones for flooring are cement (35%) and earth and sand (34%). Earth and sand are the most commonly used materials in rural households (51%), and cement is more common in urban households (50%) [7].

Punjab is the most populous province of Pakistan. As the province’s population has increased very fast, many people live in poor housing conditions. The Multiple Indicator Cluster Survey, 2017–2018 revealed that 31% of households consist of *kacha* (semi-constructed) floor, 5% of households have *kacha* (semi-constructed) roofing, and 13% of households either have *kacha* (semi-constructed) walls/no walls or natural walls. The survey also finds that three out of four children in the province under five years of age suffer from respiratory illness in the two weeks preceding the survey [8].

Household Air Pollution is the central issue in Pakistan because of the extensive usage of solid biomass along with poor housing quality, affecting the overall respiratory health of the population. As compared to many other developing countries, the studies conducted in the context to indoor air pollution are often scarce, while some of the previous studies in Pakistan, focus on the issue usually indicate that indoor air pollution should be a public health concern, whereas a limited number of studies based on interventions [9]. One of the qualitative studies conducted in the rural area of Khyber Pakhtunkhwa, Pakistan, found that with the improvement of socioeconomic status, people still prefer traditional fuels to modern ones [10]. Similarly, another study conducted in the rural area of Punjab found that the burning of biomass in an open fire causes the problem with breathing, coughing, and chest pain in women and results in indoor and local air pollution [11].

In context to the quality of housing construction, one of the studies conducted in northern areas of Pakistan concluded that the installation of fuel-efficient “smoke-free” cookstoves with chimneys, wall and floor insulations, and roof hatch windows have reduced in-house smoke and other air pollutants by over 80%, reduction in average household fuelwood expenditure of 50% and also the incidence of acute respiratory infections (ARIs), pneumonia and other illness has decreased by 50% in people [12]. Others have found that poor housing conditions and indoor air pollution were associated with the infection diseases like malaria, influenza, typhoid fever, diarrhoea and cholera among children [13,14].

**Fig 1:**
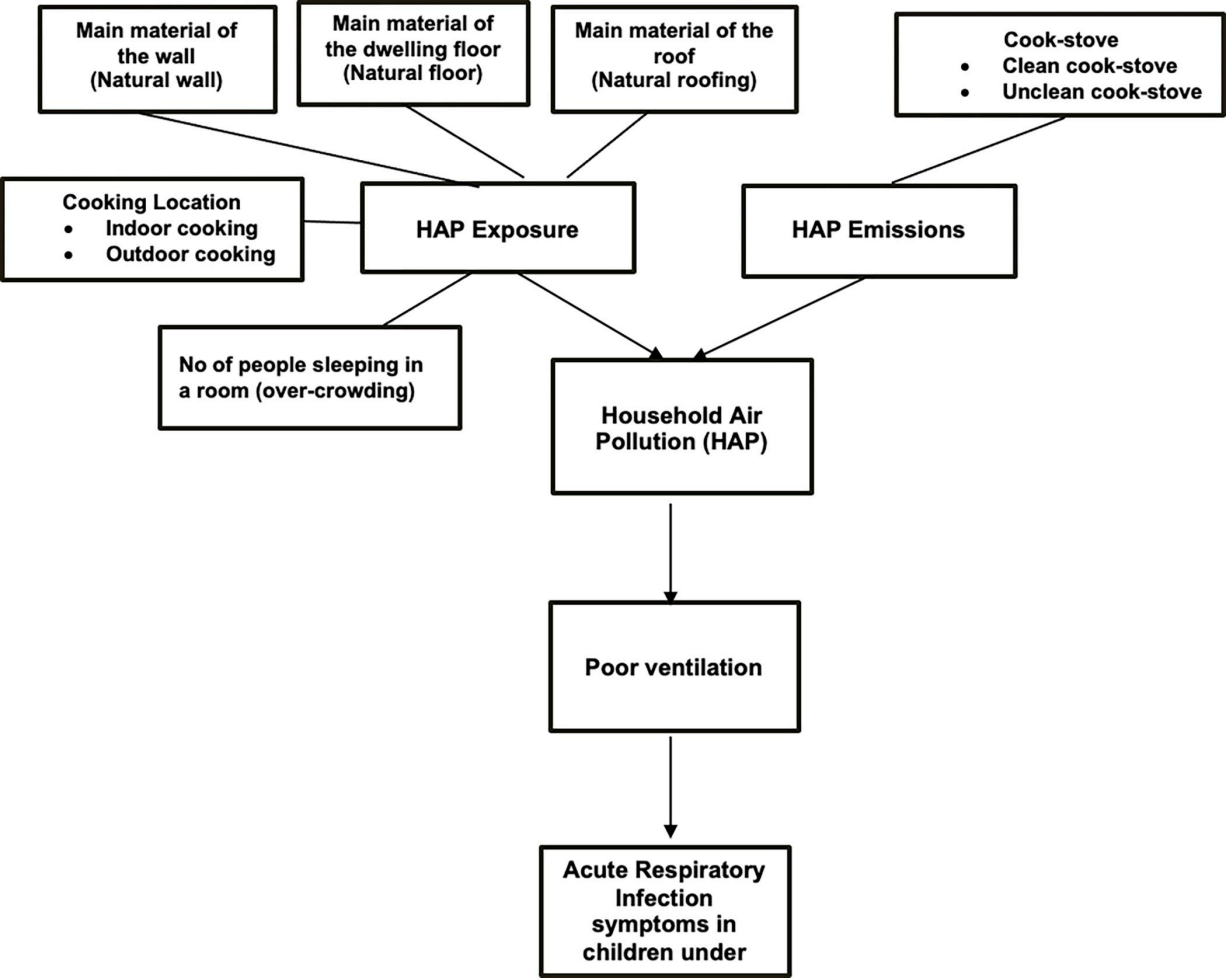
Diagrammatic representation of poor housing quality contributing to household air pollution (HAP).

However, no such study examined the impact of household infrastructure on children’s health for the overall Punjab province of Pakistan. Therefore, the purpose of the study is to investigate the housing structure as the primary determinant of Household Air Pollution (HAP) to carry out the region-wise analysis in Punjab to determine respiratory health as the primary health outcome. The conceptual framework is depicted in Fig 1. Illustrates the correlated nature of housing structure indicators considered in our study derived from the MICS questionnaire. As shown in Fig 1. The overarching hypothesis for our research is, rather than exposure to a single source of HAP, the exposure to poor housing construction material drives an association with acute respiratory infection symptoms in children. Hence, this study adds a new perspective in the literature using the above-stated determinants of HAP and children’s health endpoint as the primary outcome of interest. The study aims to provide evidence to policymakers and other stakeholders who can improve children’s health through better quality housing.

## Methods

**Fig 2:**
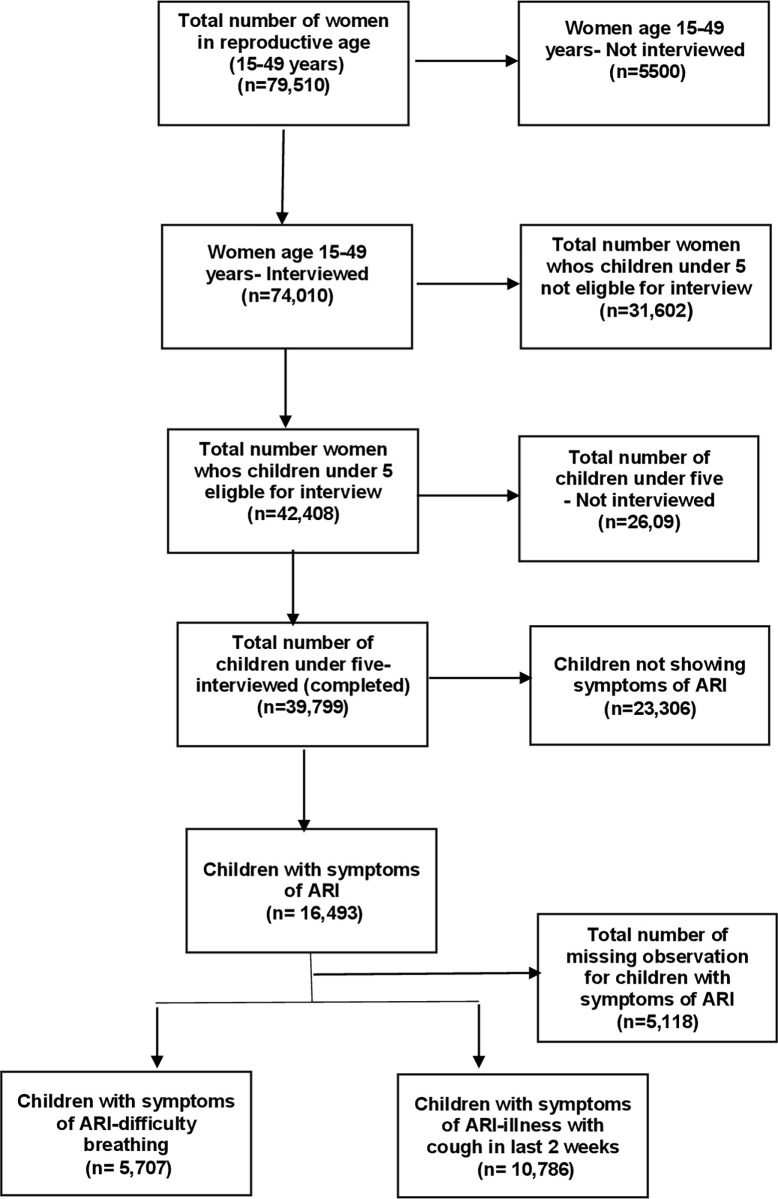
Description of study data (MICS 2017–2018).

This study uses the Multiple Indicator Cluster Survey (MICS) data from 2017 to 2018. This survey is conducted by the Bureau of Statistics, Punjab, with technical support of the United Nations Children’s Fund (United Nations International Children’s Emergency Fund). It is one of the largest and nationally representative cross-sectional household survey conducted in Pakistan, with a sample size of 53,840 households (2692 clusters) and a response rate of 97.9%. It provides updated monitoring data on women and children. A total of 42,408 children under the age of 5 were surveyed. The study sample comprises children under the age of five, reporting symptoms of ARI in the two weeks preceding the survey. These children belong to the rural and urban areas of Punjab, Pakistan [15]. The detail of the data description is shown in Fig 2.

The MICS Punjab 2017–2018 uses a two-stage stratified random sampling technique. The sampling frame of Population Census, 2017 was used. For Punjab, the sampling frame consists of 36 administrative districts. Rural and urban areas were identified as the main sampling strata within each region. A specific number of census enumeration areas were selected in the first phase, whereas, in the second phase, a household-level sampling was carried out. Twenty-five households were drawn in each sample enumeration area using a systematic sampling procedure. A random systematic sampling procedure was used to select both the enumeration areas and households. Further, in these households, all women from 15 to 49 years, all men aged 15 to 49 and children under the age of five were selected. Moreover, the survey provides all the key health indicators at the national and regional levels and in rural and urban areas. Therefore, this study restricts itself to one of the health indicators i.e. the prevalence of ARI symptoms in children under five years of age as our primary dependent variable of interest and housing quality as the independent variable from households in rural and urban are in Punjab, Pakistan [8].

## Study variables

### Acute Respiratory Infections (ARI): Dependent variable

The definition of ARI used in the Multiple Indicator Cluster Survey (MICS) is based on the mother’s perception either child has a cough, is breathing faster than usual with short, quick breaths or is having difficulty breathing excluding children that had only a blocked nose [15]. In this study, the ARI symptoms are constructed in categories, i.e., illness with cough and short/rapid/difficulty breathing problems, same as the previous literature [16–18].

### Explanatory variables

Table 1 shows the survey questionnaire housing characteristics classification (MICS 2017–2018) as natural, rudimentary, and finished.

**Table 1:** Housing characteristic summary, MICS 2017–2018.

Natural	Flooring types	Roof types	Wall types
	Earth/sand, and dung	Roof, thatch/palm leaf, sod	No walls, cane/palm/trunks, dirt
**Rudimentary**	-	Rustic mat, palm/bamboo, and wood planks	Bamboo with mud, stone with mud, uncovered adobe, plywood, cardboard, and reused wood
**Finished**	Parquet or polished wood, vinyl or asphalt strips, ceramic tiles/marble/chips, cement, carpet and bricks floor	Metal/tin/t-iron/girders, wood/wooden beams, calamine/cement fiber, ceramic tiles and cement	Cement, stone with lime/cement, bricks, cement blocks and covered adobe

Table 2 shows the categorisation of natural and finished housing materials. In this analysis roof, wall, and floor categorised into; finished roof as 0 and natural roof as 1, finished wall as 0 and natural wall as 1, finished floor as 0 and natural floor as 1.

**Table 2:** Categorisation of finished and natural housing construction.

**Natural material**	**Flooring types**	**Roof types**	**Wall types**
	Earth/sand, and dung	Roof, thatch/palm leaf, sod, rustic mat, palm/bamboo, and wood planks	no walls, cane/palm/trunks, dirt, bamboo with mud, stone with mud, uncovered adobe, plywood, cardboard, and reused wood
**Finished material**	Parquet or polished wood, vinyl or asphalt strips, ceramic tiles/marble/chips, cement, carpet and bricks floor	Metal/tin/t-iron/girders, wood/wooden beams, calamine/cement fiber, ceramic tiles and cement	Cement, stone with lime/cement, bricks, cement blocks and covered adobe

The mother’s characteristics include both the mother’s education, defined as the highest level of education she received, and her age at the time of birth. For the analysis, we have categorised the mother’s education as 1 (no education), 2 (primary/middle level of education) and 3 (secondary/higher education). Moreover, the mother’s age at the time of birth is divided into three categories; age less than 20, 20–34 years and 35+ years. In addition to this, child characteristics included the variables storing the sex of a child along with their age. The former is measured in years with equal intervals, i.e., 0, 1, 2, 3 and 4, and the latter, being a dummy, stores 1 for boys and 0 for girls. Moreover, the area of residence was also incorporated in the study, with 1 standing for the rural region and 0 for the urban region. In this study, Punjab was divided into four main regions for the analysis. Punjab regions’ classification was based on the criteria identified in previous literature [19]. It was divided into; 1 (north region), 2 (central region), 3 (south region) and 4 (west region). The study uses additional household characteristics variables, including over-crowding in sleeping rooms, cooking location, and the type of cookstoves used.

If the average number of people sleeping in a room is greater than or equal to three, it is defined as over-crowding. It takes on a value of 1 if a house has one or two rooms, and 0 if a house has greater than three rooms. The MICS collects the data on the cooking done in the house, in a separate building, or outdoors. In this study, cooking location takes on the value 1 if the cooking is done outdoor or in a separate building and 0 if there is indoor cooking.

The survey gathers data on the type of cookstove mainly used in cooking. Clean cookstove was coded as 1 if its electric stove, solar stove, liquified petroleum gas (LPG)/ cooking gas stove, piped natural gas stove, biogas stove, and a liquid fuel stove. Unclean cookstove was coded as 0 if it is a manufactured solid fuel stove, traditional solid fuel stove, and three-stone stove/open fire.

## Models

Due to the nature of the dependent variable, which is defined as a dummy, the study employed the logistic regression model. A total of two main models were specified with survey weights in which dependent variables comprised health outcomes as ARI symptoms ((i) cough with rapid/short/difficult breathing, (ii) illness with cough in children under the age of five. The main independent variables are ‘main material of floor’, ‘main material of roof’, and ‘main material of wall’. The software used to conduct the analysis is STATA 16.

The estimation models for the study are:

**log(Cough) =** β0+ β1 Floor+ β2 Roof + β3Wall + β4MotherEducation + β5MotherAge + β6 ChildAge + β7 ChildSex + β8Residence+β9Region+ β10NumberOfRooms + β11TypeOfCookstove+ β12 CookingLocation+Є (1)

**log(Ill_cough) =** β0+ β1 Floor+ β2 Roof + β3Wall + β4MotherEducation + β5MotherAge + β6 ChildAge + β7 ChildSex + β8Residence+β9Region+ β10 NumberOfRooms + β11TypeOfCookstove+ β12CookingLocation+Є (2)

## Data analysis

Descriptive statistics reporting the percentage of children suffering from ARI symptoms in rural and urban areas of Punjab from 2017-to 2018 are presented in absolute numbers and percentages. Chi-square tests were performed, and the results are disaggregated by the presence of ARI symptoms and type of housing quality. Logistic regression models have examined how poor housing quality impacts children’s acute respiratory infection symptoms. For this, a total of two main models have specified which dependent variables comprised health outcomes as ARI symptoms ((i) cough with rapid/short/difficulty breathing, (ii) illness with cough) in children under the age of five. The results of these models were expressed as adjusted odds ratios with a 95% confidence interval (CI).

## Results

**Fig 3:**
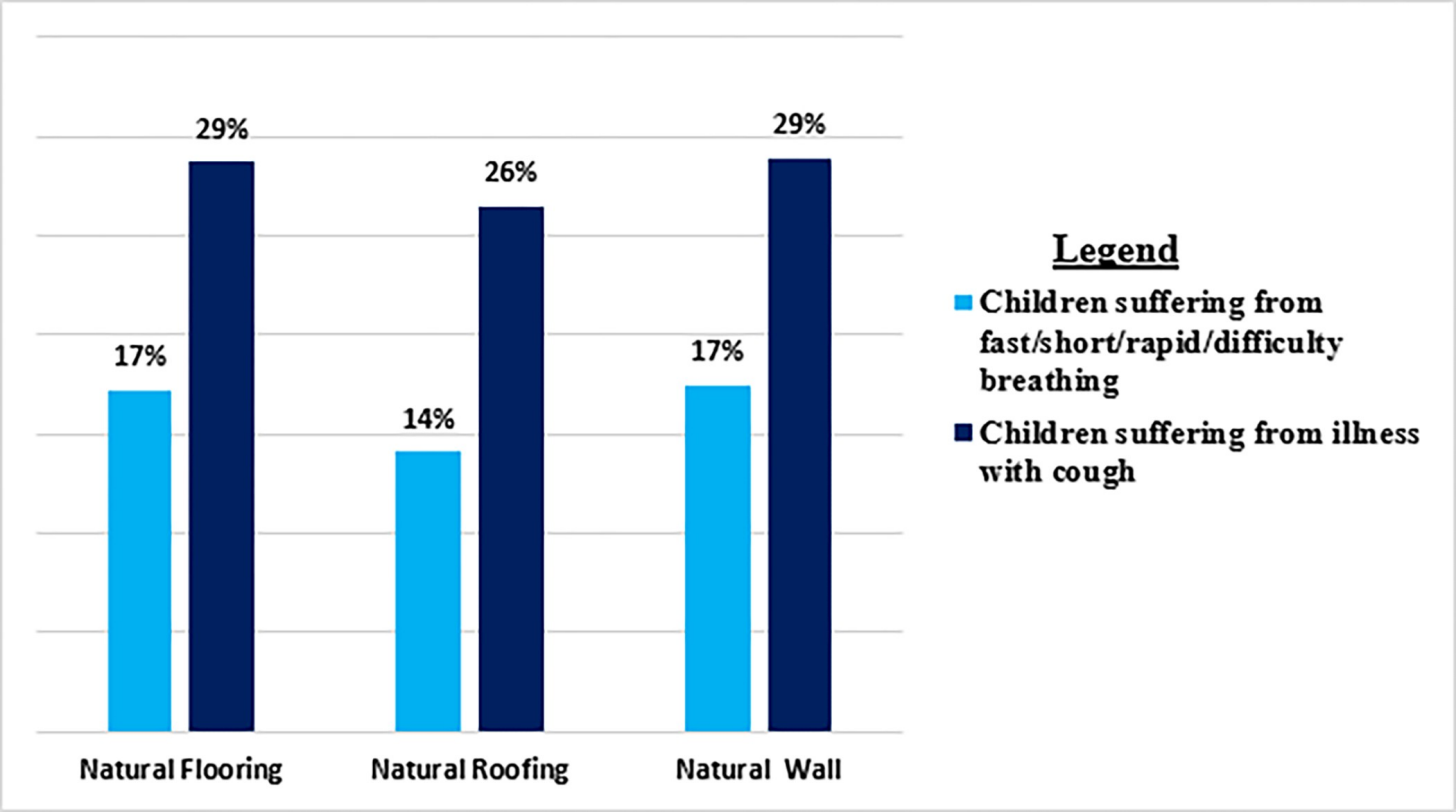
Poor housing quality and Percentage of children suffering from ARI symptoms in Punjab, Pakistan (MICS 2017–2018).

Descriptive statistics of the respondent’s housing quality and children suffering from ARI symptoms are presented in Tables 3 and 4. Around 17% of children have difficulty breathing, and 29% have an illness with cough when the floor and walls are made of natural material (Fig 3). Table 3 & Fig 3 demonstrate that 14% of children suffer from these symptoms when the housing structure comprises a natural roof compared to 86% of children.

**Table 3:** Housing quality and percentage of children with fast, short, rapid or difficult breathing in the last two weeks in Punjab, Pakistan.

Main Material of Floor	YES	NO	Total N (%)
Natural	2519 (17.20%)	12,123 (82.80%)	14,642(100%)
Finished	3184 (12.69%)	21,897 (87.31%)	25,081(100%)
**Main Material of Roof**	** **	** **	** **
Natural	400(14.06%)	2,445(85.94%)	2845(100%)
Finished	5,287(14.38%)	31,448(85.61%)	36,735(100%)
**Main Material of the wall**	** **	** **	** **
Natural	1,118 (17.44%)	5,293(82.56%)	6,411(100%)
Finished	4,567(13.76%)	28,635(86.24%)	33,202(100%)

Source: Authors own tabulation using MICS 2017–2018.

**Table 4:** Housing quality and percentage of children’s illness with cough in the last two weeks in Punjab, Pakistan.

Main Material of Floor	YES	NO	Total N (%)
Natural	4,211 (28.71%)	10,455 (71.29%)	14,666 (100%)
Finished	6,569 (26.18%)	18,519 (73.82%)	25,088 (100%)
**Main Material of Roof**	** **	** **	** **
Natural	756 (26.48%)	2,099 (73.52%)	2,855 (100%)
Finished	9,991 (27.18%)	26,764 (72.82%)	36,755 (100%)
**Main Material of the wall**	** **	** **	** **
Natural	1,851 (28.84%)	4,567 (71.16%)	6,418 (100%)
Finished	8,901 (26.79%)	24,324 (73.21%)	33,225 (100%)

Source: Authors own tabulation using MICS 2017–201.

Table 4 and Fig 3 show that around 29% of children suffer from illness with cough when the housing structure comprises natural flooring material and natural walls compared to 71% of children. Approximately 26% of children have an illness with cough while living in a house with natural roofing (Fig 3).

Table 5 shows the adjusted results of model 1 and 2. The results in model 1 show that for children under the age of five, the odds of having ARI symptoms (rapid/short/difficult breathing) are higher by 18% (OR: 1.183, CI: 1.078–1.298) for those who live in homes with natural flooring (in comparison to homes with finished flooring). However, for those who live in homes with natural walls (in contrast to finished walls), the odds of children having ARI symptoms (rapid/short/difficult breathing) are lower by 13% (OR: 0.873, CI: 0.787–0.97). Amongst children under the age of five, with a one-year increase in the child’s age, the odds of children having ARI symptoms (rapid/short/difficult breathing) are lower by 11% (OR: 0.89, CI: 0.871–0.908).

**Table 5:** Association between household characteristics, mother characteristics, child characteristics, region, residence, and under-five ARI symptoms.

Variables	Model 1: Rapid/Short/Difficult Breathing		Model 2: Illness with Cough	
	Odds Ratio(95%CI)	P value	Odds Ratio(95%CI)	P value
N	41,107		41,134	
**Material of Floor**				
Finished Flooring	1	.	1	.
Natural Flooring	1.183 (1.078 to 1.298)	0	1.04 (0.964 to 1.123)	0.31
**Material of Roof**				
Finished Roofing	1	.	1	.
Natural Roofing	0.907 (0.791 to 1.04)	0.162	0.987 (0.882 to 1.104)	0.817
**Material of Wall**				
Finished Wall	1	.	1	.
Natural Wall	0.873 (0.787 to 0.97)	0.011	0.886 (0.809 to 0.971)	0.009
**Child’s Gender**				
Girl	1	.	1	.
Boy	1.1 (1.037 to 1.168)	0.002	1.067 (1.017 to 1.119)	0.008
**Child’s Age**				
Child’s Age	0.89 (0.871 to 0.908)	0	1.019 (1.002 to 1.035)	0.025
**Area**				
Urban	1	.	1	.
Rural	1.078 (0.97 to 1.198)	0.161	0.959 (0.875 to 1.051)	0.372
**Mother’s Education**				
No education	1	.	1	.
Primary/Middle	1.081 (0.994 to 1.177)	0.07	1.174 (1.097 to 1.257)	0
Secondary/Higher	0.93 (0.839 to 1.031)	0.168	1.032 (0.951 to 1.121)	0.448
**Mother’s Age**				
Less than 20	1	.	1	.
20–34	0.979 (0.867 to 1.105)	0.729	0.985 (0.89 to 1.089)	0.768
35+	0.938(0.811 to 1.085)	0.387	0.993 (0.88 to 1.121)	0.908
**No of rooms**				
1–2 rooms	1	.	1	.
Greater than and equal 3 rooms	0.80 (0.74 to 0.870)	0	0.84 (0.787 to 0.896)	0
**Type of Cookstove**				
Clean Cookstove	1	.	1	.
Unclean Cookstove	1.12 (1.004 to 1.248)	0.042	1.012 (0.928 to 1.105)	0.783
**Cooking Space**				
Indoor Cooking	1	.	1	.
Outdoor/separate building	1.094 (1.01 to 1.186)	0.028	1.085 (1.017 to 1.157)	0.013
**Regions**				
North	1	.	1	.
South	0.682 (0.591 to 0.787)	0	0.737 (0.653 to 0.831)	0
West	1.296 (1.117 to 1.504)	0.001	1.159 (1.019 to 1.318)	0.025
Centre	0.593 (0.521 to 0.675)	0	0.659 (0.59 to 0.735)	0
Constant	0.197 (0.16 to 0.241)	0	0.369 (0.311 to 0.438)	0

For the mothers with primary/middle educational levels, the likelihood of ARI symptoms (rapid/short/difficult breathing) in their children is lower (compared to the uneducated mothers). However, the coefficients of Secondary/Higher educational level are not statistically significant. For those who live in homes with equal and greater than three rooms for sleeping (in comparison to 1–2 rooms in a household), the odds of children having ARI symptoms (rapid/short/difficult breathing) are lower by 20% (OR: 0.80, CI: 0.74–0.870). Unclean stoves and outdoor/separate buildings for cooking are associated with higher ARI symptoms (rapid/short/difficult breathing) than the clean stove and the same building for cooking, respectively.

Under-five children in the western regions had a higher likelihood of ARI symptoms than their northern counterparts. While in the southern and central areas, the possibility of having ARI symptoms is lower than their counterparts in the north. The results show that the odds of children under the age of five having ARI symptoms (rapid/short/difficulty breathing) are higher by 29% (OR: 1.296, CI: 1.117–1.504) for those who live in the western region of Punjab (in comparison to northern Punjab). Likewise, the odds of children having ARI symptoms (rapid/short/difficult breathing) are lower by 32% (OR: 0.682, CI: 0.591–0.787) and 41% (OR: 0.593, CI: 0.521–0.675) for southern and central Punjab (in comparison to northern Punjab). The results of model 2 show that for children under the age of five, the odds of having ARI symptoms (illness with cough) are lower by 12% (OR: 0.886, CI: (0.809–0.971) for those who live in homes with natural walls (in comparison to homes with finished walls).

Regarding the child’s gender, the results align with model 1, i.e., the boys are more likely to show ARI symptoms (illness with cough) than girls. Amongst children under the age of five, with a one-year increase in the child’s age, the odds of children having ARI symptoms (illness with cough) are higher by 1.9% (OR: 1.019, CI: 1.002–1.035). The results related to the mother’s education variable are aligned with model 1- i.e. (in comparison to the uneducated mothers) for the mothers with primary/middle educational level, the likelihood of ARI symptoms (illness with cough) in their children is lower. For those (less than age five) children who live in homes with more than three rooms (in comparison to rooms), the odds of children having ARI symptoms (illness with cough) are lower by 16% (OR: 0.84, CI: 0.787–0.896). Outdoor/separate buildings for cooking are associated with higher ARI symptoms (illness with cough) than cooking in the same building. These regional differences remained consistent between both models. The results show that the odds of children under the age of five having ARI symptoms (illness with cough) are higher by 15.9% (OR: 1.159, CI: 1.019–1.318) for those who live in the western region of Punjab (in comparison to northern Punjab). Likewise, the odds of children having ARI symptoms (illness with cough) are lower by 32% (OR: 0.737, CI: 0.653–0.831) and 41% (OR: 0.659, CI: 0.59 to 0.735) for southern and central Punjab (in comparison to northern Punjab).

## Discussion

Using the logistic regression model, this study addresses the gap in the literature regarding housing quality, household characteristics, child characteristics, mother characteristics, residence, region and their relationship with children’s respiratory symptoms in the Punjab Pakistan. In this study, we find that (a) residing in homes with natural flooring and wall, (b) cooking in outdoor/separate buildings, (c) unclean cookstoves significantly increase children under-five probability of experiencing ARI symptoms. Our findings show that the odds of children under five having ARI symptoms (rapid/short/difficulty breathing) are higher by 18% (OR: 1.183, CI: 1.078–1.298) for those living in homes with natural flooring. This is because the dirt floor is associated with poor health conditions among children under five [20,21], whereas the cemented floor reduces respiratory infections [21]. For the household outdoor cooking location in the presence of unclean stoves shows that children were more likely to be affected by ARI symptoms as compared to children living in a household with indoor cooking activities. Surprisingly, the results of cooking location contradict the previous studies in developing countries [22,23]. This is probably due to the polluting cookstoves coupled with the outdoor cooking activities and poor ventilation associated with the children suffering from respiratory symptoms compared to the cooking done in a household [24]. This is an area of future research.

The results of our study also share several similarities with the previous research conducted in different countries. In Lao PDR [25], the study demonstrates the statistically significant association between child respiratory health outcomes and housing material of the floor, wall. In contrast, roofing material was not a significant factor in child health. Similarly, a community-based study conducted in the urban slums of Assam, India [26] and Nigeria [27] shows a strong statistical association between occurrences of ARI symptoms in children under the age of five living in poor housing materials. In the context of maternal education, the study identifies the odds of ARI among children as substantially high among children of mothers with primary/middle education compared to no education. The results are very much similar to previous research conducted in Bangladesh [28], Nigeria [29] and Indonesia [30]. This might be the case as educated mothers are more likely to get employed and left alone their children in someone else place whose lifestyle may influence respiratory symptoms among children. Moreover, our finding in the context of overcrowding contradicts the study in Brazil [31]. The lower likelihood of ARI symptoms among children sleeping in a household with more than three rooms might be because of a window that helps in ventilation.

Whereas the findings in terms of gender remain in line with the previous research conducted in Bangladesh [28] and in India [32], the results show a significant association with boys more likely to get ARI symptoms compared to girls. This may be related to different behaviour as boys are more active and usually spend more time outdoor, increasing their risk of ARI symptoms. In the domain of residential factors, the findings of ARI symptoms are not statistically significant in the study. These results are in line with the previous research conducted in Bangladesh, which shows a place of residence has no influence on a child’s respiratory symptoms [33]. In contrast, the study conducted in India offers a higher prevalence of ARI symptoms among children under the age of five from urban areas than in rural areas due to overcrowding settings, the difference in socioeconomic factors, and cultural factors in these urban areas [34].

We also found the likelihood of ARI symptoms is higher among children under five in the western region of Punjab than in other areas. The literature revealed that the western part of Punjab is bordered on its far north and northwest by the Khyber Pakhtun Khawan, the snow-capped Hindu Kush mountains, and the high Karakoram Range [35]. The increasing dry seasonal pattern of western Punjab [35] might be the reason for the increased odds of children suffering from ARI symptoms. During dry seasons, pathogens are often blown around with dust, thereby increasing the risk of infections in children [27].

The findings of the studies add to the growing body of literature on housing quality and its impact on child’s health. The uniqueness of the study is its focus on the independently access measure of housing quality and its association with the ARI symptoms among under-five children in Punjab between 2017–2018, whereas its strength lies in the use of Multiple Indicator Cluster Survey (MICS) data which is nationally representative and has been through a validation process; therefore, its results can be generalisable.

Our study is not without its limitations. First, it was based on the self-reporting information from mothers about their children. This may be a source of recall bias information as the data of ARI symptoms in children are not collected by MICS from the clinical examination rather than from the mother’s reporting on the children experiencing symptoms that can vary from mother to mother based on their education, understanding and perception. Moreover, this study could not control for many other child characteristics such as vaccination status, birth order and child breastfeeding status due to the unavailability of data. Another limitation was our inability to include the chimney variable among explanatory variables in the primary regression model due to missing values. Further, this research is limited to district-wise data only in one province of Pakistan; therefore, it is likely that the study will produce different results for the other provinces depending upon their socio-economic conditions.

### Policy implications

In Pakistan, on average, 14% of children under the age of five suffer from Acute Respiratory infections (ARIs), making it a leading cause of childhood morbidity and mortality [36]. On the other hand, housing in Pakistan is stated to be unaffordable due to lack of housing finance, poor quality and overcrowded, where the majority of 30% to 50% of urban dwellers lives in “slums” or “katch-abadi” (temporary settlements) [7]. Our foremost policy recommendation to the government is to replace the poor quality housing material. For this, the government should replace the natural floors with cement floors, similar to Mexico’s anti-poverty conditional cash transfer program (Piso Firme), which replaces dirt floors with cemented ones in low-income families, which eventually has a positive impact on child health [20]. Also, through the government’s favourable house building finance institutions/model, one can promote access to affordable housing options. Behavioural strategies of encouraging quality material during housing construction with health education sensitise to ventilation are another policy interventions the government could undertake to reduce the adverse health outcomes in children. A second policy recommendation would be the targeted distribution of clean cookstoves at affordable pricing.

The housing problem is not a problem that architects can solve alone. Still, it’s a multi-faceted problem that can only be solved through raising living standards, improving employment opportunities and urban regulations [5], whereas formulating and implementing strategies through collaboration will combat local issues and problems at micro-level.

## Supporting information

S1 Data Do File (STATA) of the dataset used for analysis of the current study. (DO)
